# Airborne fungi as indicators of ecosystem disturbance: an example from selected Tatra Mountains caves (Poland)

**DOI:** 10.1007/s10453-017-9498-y

**Published:** 2017-09-27

**Authors:** Wojciech Pusz, Maria Król, Tomasz Zwijacz-Kozica

**Affiliations:** 1 Department of Plant Protection, Wroclaw University and Environmental and Life Sciences, Grunwaldzki Sq. 24A, 50-363 Wrocław, Poland; 2 Tatra National Park, Kuźnice 1, 34-500 Zakopane, Poland

**Keywords:** Speleomycology, Aeromycology, Monitoring, Caves, Biodiversity

## Abstract

We report on the determination of the spore concentration and the species composition of the airborne fungi in selected caves of the Tatra Mountains, Poland. The following caves were surveyed: Mylna, Obłazkowa, Mroźna, Zimna and Naciekowa. The sampling was carried out in July 2015 and in January 2016. The aeromycological analyses were performed with the impact method, using the Air Ideal 3P apparatus and potato dextrose agar (PDA, Biocorp) culture medium. In the course of the July 2015 analysis, 17 species of fungi were isolated and 11 species were isolated in January 2016. In Mylna and Naciekowa caves, the dominant species were *Cladosporium cladosporioides* and *Stachybotrys cylindrospora*. In Obłazkowa cave, *Rhizoctonia* predominated and in Zimna cave—the colonies of the yeast-like fungi, along with *S. cylindrospora*. In Mroźna cave, *Penicillium notatum* was the most abundant taxon. In the winter time, in the majority of the caves *Penicillium* spp. predominated, with the exception of Mroźna and Naciekowa caves where *Aspergillus niger* was dominant. We propose that aeromycological monitoring be performed regularly in the following caves: Mroźna, Naciekowa and Zimna.

## Introduction

Fungi are an important part of the ecosystems of the underground environments, where they play the role of decomposers or exist as parasites. They occur predominantly on organic matter, such as animal feces, wood or carcasses (Nespiak 1970; Shapiro and Pringle 2010; Taylor et al. 2012; Vanderwolf et al. 2013). Although the low temperature and low nutrient availability make underground environments unfavorable for the development of fungi, these organisms may survive there (Bastian et al. 2000). Most often, fungi occur in underground spaces as spores, imported there with air currents, water, or transported by cave-inhabiting animals such as bats or arthropods, and by human visitors (Schabereiter-Gurtner et al. 2004; Fernández-Cortès et al. 2011). They constitute food for invertebrates and are very important components of the ecology of caves and other environments (Chelius et al. 2004; Docampo et al. 2011).

The fungal taxa most often encountered in caves are those belonging to the genera of *Aspergillus*, *Penicillium*, *Mucor*, *Fusarium*, *Trichoderma* and *Cladosporium* (Gadd 2007). The spores most often present in the air inside the caves belong to *Penicillium* spp. and *Cladosporium* spp., as well as to *Alternaria* spp. (Saíz-Jiménez 2012; Pusz et al. 2014, 2015). Our survey has demonstrated that the incidence of fungi became reduced in winter time, which is in agreement with the results of other authors (Li et al. 2008; Nováková 2009).

It has been ascertained in many studies that the density of fungi that occur in caves correlates tightly with the number of visiting tourists and bats, and with the air movement (Pusz et al. 2014, 2015). Moreover, some authors claim that it is the appearance of humans in caves that initiates the process of the cave colonization by microorganisms including fungi, and that it contributes to the microclimate change within the cave environment, mostly to the increase in temperature and atmospheric CO_2_ concentration (Fernández-Cortès et al. 2011; Porca et al. 2011). Consequently, the growing number of visiting tourists may be accompanied by an increase in the number of fungal colonies, as well as in the species count, in a cave, which negatively affects the biological balance of the cave ecosystem, contributing to biochemical changes taking place on the rock surface (Gadd 2007; Mulec 2008).

Another important aspect of the presence of fungi, and their morphological structures, in caves and in other underground environments, is their potentially negative influence on the human health. Fungi may be the cause of serious infectious diseases and allergies, e.g., the taxa of *Aspergillus* spp. are capable of causing infections in humans and other mammals, as well as pulmonary aspergillosis (Cabral 2012).

In the caves and underground spaces that are made commercially accessible for tourists, the incidence of fungi is manyfold higher compared to those, which are not open to visitors (Pusz et al. 2014, 2015). Many researchers argue that caves deserve regular environmental monitoring, including the assessment of the incidence of fungi and of the concentration of their spores, aimed at tracing the changes that take place in cave microenvironments under the influence of external factors such as tourist traffic (Porca et al. 2011; Pusz et al. 2014).

**Fig. 1:**
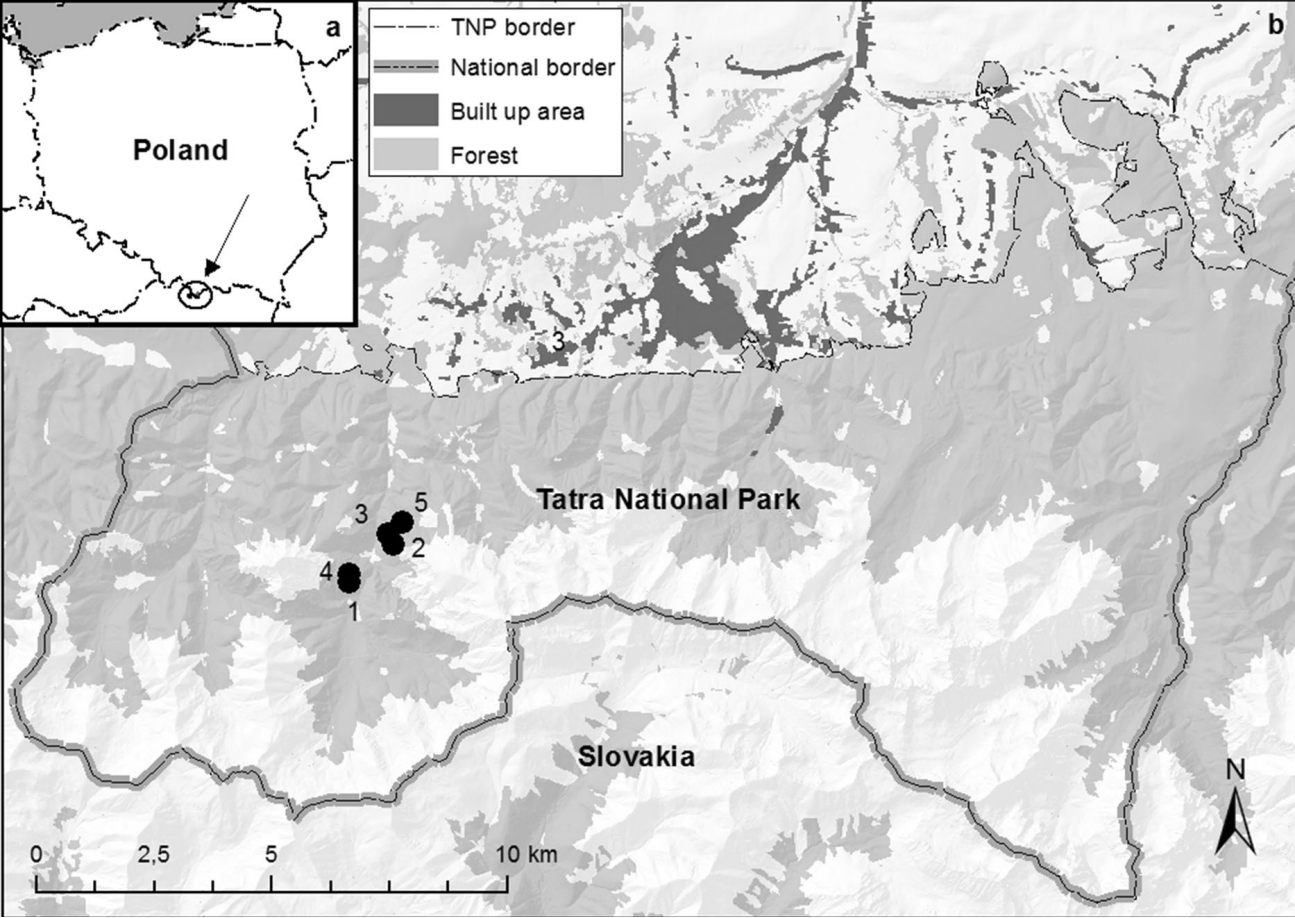
**a** Location of study area in Poland (Central Europe). **b** Location of sampled caves in the Tatra National Park: 1—Obłazkowa cave; 2—Zimna cave; 3—Mroźna cave; 4—Mylna cave; 5—Naciekowa cave

The objective of the presented research was to determine the spore concentration and the species composition of the airborne fungi in selected caves of Tatra Mts: Mylna, Obłazkowa, Mroźna, Zimna and Naciekowa caves (Fig. 1).

## Materials and methods

### Study site

The study was located in the Kościeliska Valley, in the western part of the Tatra National Park (TNP, southern Poland). There are more than 800 caves known in the TNP. Most of them are located in Kościeliska Valley (Table 1).

**Table 1:** Characterizations of the surveyed caves

Cave	Length	Altitude and exposure of the entrance	Number of visitors	Incidence of bats	Climate
Obłazkowa	120 m	1098 m asl, SSE	Several thousands of tourist per year (open year round)	Very rare	Dynamic
Zimna	5335 m	1: 1120 m asl, WSW 2: 1260 m asl, SW	Several hundreds of spelunkers per year (open year round, but only for spelunking)	Abundant	Dynamic up to 500 m from the lower entrance, deeper inside static
Mroźna	511 m	1: 1100 m asl, E 2: 1112 m asl, SSW	130–160 thousands of tourist per year (open from May till September)	Rare	Static (entrances closed by gates)
Mylna	approx. 1300 m	approx. 1098 m asl, SSE, E i NE	Several thousands of tourist per year (open year round)	Rare	Dynamic
Naciekowa	1210 m	1: 1188 m asl, SW 2: 1180 m asl, S 3: 1199 m asl, SW	Several dozens of visitors per year (cave open only for scientific investigation, but illegal visits occur too)	Abundant	Dynamic

### Sampling

The survey was carried out in July 2015 and in January 2016. The aeromycological analyses were performed with the impact method, using Air Ideal 3P apparatus and potato dextrose agar (PDA) medium culture (Biocorp) using the air samples of 50, 100 and 150 l, in three replicates, which were taken in each cave. The sampling device had been always placed at 1.5 m above the ground level. The PDA medium was incubated in room temperature (22 °C) for 7 days. After that time, the colonies appearing on the medium were counted, and the organisms were determined taxonomically to the species level, based on their morphological traits (Pitt and Hocking 2009; Watanabe 2011). The number of the colony-forming units (CFU) per 1000 l (1 m^3^) of the air was calculated. The number of fungal colonies on particular Petri dish was transformed into the CFU per 1 m^3^ of the air according to the formula:

$$ X = \left( {a \times 1000} \right)/V $$

where *a* denotes the total number of colonies cultured on the Petri dish from the sample of the atmospheric air and *V* volume of the sampled air in liters.

The data were compared using biodiversity indices: Margalef’s index, Simpson’s index, Shanon–Wiener index and Pielou index (Begon et al. 2006).

## Results

In the course of the July 2015 analysis, 17 species of fungi were isolated altogether, whereas 11 species were isolated in January 2016 (Tables 2, 3).

**Table 2:** Airborne fungi isolated from Tatra Mts. caves in summer 2015 (CFU/m^3^) and biodiversity indices

Fungal taxa	Kościeliska Valley	Obłazkowa cave	Zimna cave	Mroźna cave	Mylna cave	Naciekowa cave
*Alternaria alternata*	67	6			12	24
*Alternaria botrytis*	6					
*Botrytis cinerea*	17					13
*Cladosporium cladosporioides*	213		11	12	20	145
*Cladosporium herbarum*	20				6	
*Drechslera sorokiniana*	19					
*Fusarium culmorum*	20					
*Fusarium oxysporum*	5					
*Penicillium citrinum*	7					19
*Penicillium melagrinum*		6			26	42
*Penicillium notatum*	21			22		6
*Penicillium* spp.			5	7	6	
*Rhizoctonia* spp.		47	5	10		
*Sclerotium rollfsi*			5	5	5	
*Stachybotrys cylindrospora*		12	17		53	
*Trichoderma harzianum*	26					
*Trichotecium roseum*		7				
Yeast colonies			21	21		
White non-sporulating colonies					40	4
Total number of CFU	421	78	64	77	168	253
Number of species	11	5	6	6	7	7
Margalef’s index	1.655	0.918	1.063	0.994	1.237	0.906
Simpson’s index	3.374	2.459	3.812	3.910	3.971	2.591
Shannon–Wiener index	1.699	1.204	1.466	1.480	1.614	1.281
Pielou index	0.709	0.748	0.911	0.920	0.830	0.715

**Table 3:** Airborne fungi isolated from Tatra Mts. caves in winter 2016 (CFU/m^3^) and biodiversity indices

Fungal taxa	Kościeliska Valley	Obłazkowa cave	Zimna cave	Mroźna cave	Mylna cave	Naciekowa cave
*Alternaria alternata*	13	33				
*Asoergillus flavus*		6				
*Aspergillus niger*	20	13	79	134		77
*Botrytis cinerea*						
*Cladosporium cladosporioides*		13		6		
*Fusarium oxysporum*		33				
*Penicillium citrinum*		20		6		
*Penicillium chrysogenum*	20		80	74	26	18
*Penicillium melagrinum*	26	21	40	201	73	46
*Penicillium notatum*	26	33	73	20		
*Penicilluum urticae*			46			
White non-sporulating colonies	20					12
Total number of CFU	125	172	318	441	99	153
Number of species	6	8	5	6	2	3
Margalef’s index	1.036	1.522	0.694	0.821	0.218	0.404
Simpson’s index	5.742	7.551	4.663	3.024	1.632	2.376
Shannon–Wiener index	1.768	2.094	1.571	1.277	0.576	0.959
Pielou index	0.987	0.953	0.976	0.713	0.831	0.874

During the summer of 2015, 11 species of fungi were identified in the atmospheric air in the Kościeliska Valley, and their concentration amounted to 421 CFU/m^3^. The predominant species was *Cladosporium cladosporioides*. The second dominant was *Alternaria alternata*, followed by *Trichoderma harzianum* and by the taxa belonging to the genera of *Penicillium* and *Fusarium*. At the same time, only five species were found in Obłazkowa cave, six species in Zimna and Mroźna caves and seven species in Mylna and Naciekowa caves. The highest concentrations of the fungi were detected in Naciekowa and Mylna caves (253 and 168 CFU/m^3^, respectively). It was *C. cladosporioides* and *Stachybotrys cylindrospora* that had apparently dominated in the former or in the latter cave, respectively. In the remaining caves, the CFU/m^3^ values oscillated between 64 and 78. In Obłazkowa, the species of the genus *Rhizoctonia* prevailed and in Zimna cave—the colonies of the yeast-like fungi along with *S. cylindrospora*. In Mroźna cave, in turn, *Penicillium notatum* had outnumbered the other taxa.

The calculated values of the Shannon–Wiener, Pielou, Simpson and Margalef’s biodiversity indices justify the conclusion that, of all the Tatra Mts. caves selected for the survey, the highest diversity of the airborne fungi can be found in Mylna and Mroźna caves. On the other hand, the diversity measured in the summer time is at its lowest level in Obłazkowa and Naciekowa caves.

In winter time, six species of fungi were detected in the air of Kościeliska Valley, with their spore concentration amounting to 125 CFU/m^3^. The majority of the taxa sampled there belong to *Penicillium* genus: *P. chrysogenum*, *P. meleagrinum* as well as *P. notatum*. The CFU/m^3^ values in the surveyed caves varied widely between 99 in Mylna cave, through 153–172 in Naciekowa and Obłazkowa caves to 318 and 441 CFU/m^3^ in Zimna and Mroźna caves, respectively. Except for Mroźna and Naciekowa caves, where *Aspergillus niger* was the dominant taxon, in the remaining objects the species of the genus *Penicillium* were prevailing.

Based on the Shannon–Wiener, Pielou, Simpson and Margalef’s biodiversity indices, that during the winter season the diversity of the airborne fungi is the highest in Obłazkowa cave. In turn, the lowest fungal diversity in winter time is found in Mylna and Naciekowa caves.

## Discussion

The results obtained in our survey correspond to the work of other authors in terms of the species composition of the fungal associations of the underground environments, CFU concentration and the species diversity across the seasons (Nováková 2009; Docampo et al. 2011; Porca et al. 2011; Vanderwolf et al. 2013; Pusz et al. 2014, 2015; Kokurewicz et al. 2016; Ogórek et al. 2016).

Bats as much as other animals (Vanderwolf et al. 2013; Kokurewicz et al. 2016) are listed as the main suppliers of the organic matter to the caves and other underground spaces and, by the same token, as suppliers of nutrients to the fungi residing there. Humans, however—the tourists, explorers and speleologists—are claimed to be even more important in this respect (Porca et al. 2011). Too high a number of the visitors may trigger ecosystem disturbance leading to irreversible, negative changes in the environment that are threat to the entire cave ecosystem (Bastian et al. 2000; Dupont et al. 2007; Fernández-Cortès et al. 2011). It therefore appears that monitoring of caves and other underground environments is an issue of critical importance, and that guidance should be elaborated, taking into consideration all parts of the underground ecosystem as an indivisible, complex assembly (Porca et al. 2011; Cabral 2012; Donato et al. 2014).

Caves are natural habitat included in Natura 2000 network of protected areas. The Natura 2000 Network will consist of sites designated by the Member States of the European Union, under the Habitats and Birds Directives. Many of these sites, like meadows, forests and caves, need an appropriate management to maintain a favorable conservation status (Ostermann 1998). Caves are coded 8310 as “not open to the public or not routinely exploited for tourism.” In the detailed guide for the methodology of natural habitat monitoring, Mróz (2015) recommends primarily the observation of the abiotic natural phenomena, such as microclimate and hydrological factors. Yet the author also emphasizes the importance of the anthropopressure and its assessment, as well as of the estimation of the density and structure of the overwintering chiropterofauna and of the incidence of invertebrates (Mróz 2015). It seems, however, that the monitoring guidance put forward is partly inadequate, and that the mycological monitoring should be carried out in caves and in other underground environments, the more that this recommendation complies with the suggestions made by some other authors (Donato et al. 2014). Fungi are one of the groups of bioindicator organisms which may point to changes taking place in the environment. The observation of their incidence may therefore facilitate answers to questions concerning stability of the underground ecosystems. Porca et al. (2011) had proposed the critical thresholds of CFU concentration *per* m^3^ and suggested that they are used as indicative for the status of a cave:

- *Category 1* Underground environments not endangered by fungal infestation (CFU concentration < 50 per m^3^);
- *Category 2* Underground environments vulnerable to fungal infestation (CFU concentration 50–100 per m^3^). Periodical aeromycological monitoring necessary;
- *Category 3* Underground environments threatened by fungal infestation (CFU concentration 150–500 per m^3^). Periodical aeromycological monitoring necessary, measures to restrict importation of fungal material from the outside necessary, e.g., limited visit frequency, ventilation systems and air lock;
- *Category 4* Underground environments affected by fungal infestation (CFU concentration 500–1000 per m^3^). Possible damage to the natural forms (like stalactite) of the cave and to artefacts, including historical artefacts;
- *Category 5* Underground environments irreversibly destroyed by fungal infestation (CFU concentration > 1000 per m^3^).

Underground environments classified in the classes 4 and 5 should be subject to strict monitoring. All possible measures should also be taken in order to prevent incidence of such extreme concentrations of airborne spores.

Based on the analysis of the CFU concentrations inside the five Tatra Mts. caves surveyed, and considering the fact that the exploration of the caves that have not been made accessible for the mass tourist traffic is actually taking place, one may assume that, among the caves made only accessible for spelunking the Zimna cave can be characterized as threatened. In winter season, the concentration of the CFU/m^3^ attained 318 in that cave; thus, according to the classification by Porca et al. (2011) it would be classified as category 3. Similar is the situation of Naciekowa cave, in which the concentration of 153 CFU/m^3^ was recorded in winter time and of 253 CFU/m^3^—in summer. In Mroźna cave, the relatively high CFU values (441) were recorded in winter season, which may reflect the fact that this non-ventilated cave had been closed completely after the end of the tourist season, i.e., following the relatively long period of accumulation of the spores and other fungal organs.

Taking into consideration of the fact that fungi constitute a potential threat to cave ecosystems and are, at the same time, indicative for the changes taking place in such ecosystems, it seems that the aeromycological monitoring should be carried out in caves, and that it should be performed in two seasons of the year—in winter and in summer. This would possibly allow to assessment of risk stemming from making the particular caves accessible to tourists and spelunkers and, by the same token, might lead to implementation of protective measures, starting from more efficient ventilation (e.g., Mroźna cave) through to the curtailment of the tourist traffic in the caves that are made only accessible to spelunkers. Unfortunately, the feasibility of monitoring and controlling illegal visits to the caves is negligible. Moreover, one should keep it in mind that fungi are not only a threat to the caves, but also to their visitors. Although the CFU/m^3^ values determined inside the surveyed caves do not, typically, pose a hazard to humans, individuals with compromised health status may experience negative effects of their staying longer inside the caves where CFU values exceed 500 (Cabral 2012).

## Conclusions

Analyzing the obtained results, it can be concluded that the present tourist traffic in the Tatra Mts. caves does not negatively affect the species composition and concentration of CFU in the air. The environment of caves is the ecosystem that can respond to changes taking place in different ways and at different rates. It seems that recurring conduct aeromycological monitoring may indicate a subtle change: (i) have taken place in the ecosystem, (ii) currently taking place in the caves, (iii) begin to occur in caves. This type of observations should be carried out in caves without any restrictions on tourists visiting (e.g., Mroźna cave). However, it should be borne in mind that any caves or other underground’s environment should treat the individual as the conditions which prevail in them can eliminate the negative impact of tourism, for example by the movement of air. However, in some cases, tourist traffic can be limited as it did in the most famous cave in the world, the cave of Lascaux.
